# Effect of coffee thermal cycling on the surface properties and stainability of additively manufactured denture base resins in different layer thicknesses

**DOI:** 10.1111/jopr.13803

**Published:** 2023-12-06

**Authors:** Gülce Çakmak, Nura Watson Asadolahi, Martin Schimmel, Pedro Molinero‐Mourelle, Canan Akay, Mustafa Borga Donmez, Burak Yilmaz

**Affiliations:** ^1^ Department of Reconstructive Dentistry and Gerodontology School of Dental Medicine University of Bern Bern Switzerland; ^2^ Division of Gerodontology and Removable Prosthodontics University Clinics of Dental Medicine University of Geneva Geneva Switzerland; ^3^ Department of Prosthodontics Faculty of Dentistry Osmangazi University Eskisehir Turkey; ^4^ Translational Medicine Research and Clinical Center Osmangazi University Eskisehir Turkey; ^5^ Department of Prosthodontics Faculty of Dentistry Istinye University İstanbul Turkey; ^6^ Department of Restorative Preventive and Pediatric Dentistry School of Dental Medicine University of Bern Bern Switzerland; ^7^ Division of Restorative and Prosthetic Dentistry The Ohio State University Ohio USA

**Keywords:** denture base, layer thickness, microhardness, stainability, surface roughness

## Abstract

**Purpose:**

To compare the effect of coffee thermal cycling on surface roughness (Ra), Vickers microhardness (MH), and stainability of denture base resins additively manufactured in different layer thicknesses with those of subtractively manufactured denture base materials.

**Materials and Methods:**

Eighty disk‐shaped specimens (Ø10×2 mm) were fabricated from two subtractively (Merz M‐PM [SM‐M] and G‐CAM [SM‐G]) and three additively (NextDent 3D+ [50 µm, AM‐N‐50; 100 µm, AM‐N‐100], FREEPRINT Denture [50 µm, AM‐F‐50; 100 µm, AM‐F‐100], and Denturetec [50 µm, AM‐S‐50; 100 µm, AM‐S‐100]) manufactured denture base materials (*n* = 10). Ra measurements were performed before and after polishing by using a non‐contact optical profilometer, while MH values and color coordinates were measured after polishing. Specimens were then subjected to 5000 cycles of coffee thermal cycling, all measurements were repeated, and color differences (ΔE00) were calculated. A linear mixed effect model was used to analyze Ra and MH data, while one‐way analysis of variance was used to analyze ΔE00 data (α = 0.05). Ra values were further evaluated according to a clinically acceptable threshold of 0.2 µm, while ΔE00 values were evaluated according to perceptibility (1.72 units) and acceptability (4.08 units) thresholds. The interaction between the material type and the time interval affected both Ra and MH (*p* ≤ 0.001). Tested materials had their highest Ra before polishing (*p* ≤ 0.029). Before polishing, AM‐F‐100 had the highest, and SM‐M and SM‐G had the lowest Ra (*p* < 0.001). After polishing and after coffee thermal cycling, SM‐G mostly had lower Ra than those of other materials (*p* ≤ 0.036). SM‐G mostly had higher MH than that of other materials before and after coffee thermal cycling (*p* ≤ 0.025). Coffee thermal cycling reduced the MH of SM‐M and increased that of AM‐S‐100 (*p* ≤ 0.024). AM‐N‐100 had higher ΔE00 than AM‐F, AM‐S‐100, and SM‐G (*p* ≤ 0.009), while AM‐F and SM‐G had lower ΔE00 than AM‐S‐50 and AM‐N‐50 (*p* ≤ 0.024).

**Conclusions:**

Polishing reduced the surface roughness of all materials, whereas the effect of coffee thermal cycling was nonsignificant. Most of the tested materials had acceptable surface roughness after polishing and after coffee thermal cycling according to the reported threshold. Layer thickness only affected the microhardness of tested additively manufactured resins, which was material‐dependent. Subtractively manufactured specimens mostly had high microhardness and that of nonreinforced subtractively manufactured resin decreased after coffee thermal cycling. When reported color thresholds are considered, all materials had acceptable color stability.

Even though dental implants are increasingly used, complete dentures and removable partial dentures are still feasible treatment modalities given medical and financial reasons.^1^, ^2^ Polymethylmethacrylate (PMMA), which is a synthetic polymer^3^ has been the preferred material for dentures due to its low cost, biocompatibility, ease of processing, and acceptable esthetics.^4^ However, conventionally manufactured PMMA is prone to polymerization‐related issues that could deteriorate after long‐term use due to increased risk of mechanical and biological complications.^5^, ^6^, ^7^ Advancements in computer‐aided design and computer‐aided manufacturing (CAD‐CAM) technologies have enabled the fabrication of complete dentures subtractively or additively.^8^, ^9^, ^10^, ^11^, ^12^, ^13^, ^14^ These digital techniques reduce the steps involved in fabrication, which also leads to reduced fabrication time and costs.^15^ In addition, nanotechnology has also been integrated into dentistry, and graphene, which is a crystalline form of carbon with a honeycomb‐shaped arrangement, is used to reinforce PMMA as nanographene‐reinforced PMMA disks are commercially available.^16^, ^17^, ^18^

Regardless of the manufacturing method, irregularities or indentations on a denture's surface may lead to biofilm adherence and denture stomatitis^19^ and previous studies have reported 0.2 µm as the threshold for plaque accumulation.^3^, ^8^, ^20^, ^21^, ^22^, ^23^, ^24^, ^25^ In addition, an increase in surface roughness may impair dentures’ esthetic appearance due to color change,^26^ which might lead to the replacement of the denture^9^, ^27^, ^28^ as color change can be an indicator of material damage and aging.^8^ Denture base materials are frequently exposed to intraoral thermal stresses caused by hot and cold beverages, which might deteriorate their surface.^21^, ^29^ The presence of a liquid medium might also diminish the mechanical properties of a denture base material as absorption of water into inter‐polymeric gaps forces polymeric chains to move away from each other.^30^ Therefore, denture base materials should be able to maintain their surface integrity and have high color stability even after long‐term use.

Printing layer thickness affects different properties of additively manufactured denture base resins that may affect their clinical longevity such as flexural strength, bacterial film adhesion, and fabrication trueness.^31^, ^32^, ^33^ Even though the mechanical and optical properties of additively manufactured denture base materials have been investigated,^5^, ^6^, ^11^, ^12^, ^16^, ^17^, ^18^, ^19^, ^30^, ^34^, ^35^, ^36^ the authors are aware of two studies on the effect of printing layer thickness on the roughness or stainability of additively manufactured resins.^13^, ^37^ However, one of those studies focused only on the surface roughness of denture base resins fabricated in different layer thicknesses,^13^ while the other one did not involve denture base resins,^37^ and neither involved a comparison with subtractively manufactured resins. Therefore, the main aim of the present study was to compare the surface roughness (R_a_), Vickers microhardness (MH), and stainability of additively manufactured denture base materials in different layer thicknesses to those of subtractively manufactured denture base materials that were either reinforced with nanographene or not, after coffee thermal cycling. In addition, the polishability of tested materials was evaluated. The null hypotheses were that: (i) material type and time interval (before polishing, after polishing, and after coffee thermal cycling) would not affect R_a_, (ii) material type and time interval (after polishing and after coffee thermal cycling) would not affect MH, and (iii) material type would not affect stainability.

## MATERIALS AND METHODS

Seven specimens per test group were deemed adequate by a priori power analysis (*f* = 0.73, 1‐*β* = 95%, *α* = 0.05) based on the results of a previous study that evaluated the surface roughness and stainability of additively and subtractively manufactured denture base materials.^17^ Ten specimens per test group were fabricated to increase the power. Two subtractively (Merz M‐PM; Merz Dental GmbH [SM‐M] and G‐CAM; Graphenano DENTAL [SM‐G]) and three additively (NextDent Denture 3D+; NextDent B.V. [AM‐N], FREEPRINT denture; Detax [AM‐F], and Denturetec; Saremco AG [AM‐S]) manufactured denture base materials were used to fabricate disk‐shaped specimens (Ø 10 mm × 2 mm) (Table 1).

**TABLE 1:** Detailed information on denture base materials tested in this study.

	Type	Chemical Composition
Merz PM (SM‐M)	Subtractively manufactured denture base resin	Polymethylmethacrylate and cross‐linked polymers based on methacrylic acid esters, colorants, residual peroxide as dibenzoyl peroxide, methylmethacrylate (MMA) may be contained as residual monomer up to max 1%
G‐CAM (SM‐G)	Subtractively manufactured nanographene‐reinforced denture base resin	Not disclosed
NextDent Denture 3D+ (AM‐N)	Additively manufactured denture base resin	Methacrylic oligomers, methacrylate monomer, inorganic filler, phosphine oxides, pigments
FREEPRINT denture (AM‐F)	Additively manufactured denture base resin	Isopropylidenediphenol peg‐2 dimethacrylate: 35%–<60% 7,7,9‐trimethyl‐4,13‐dioxo‐3,14‐dioxa‐ 5,12‐diazahexadecane‐1,16‐diyl bismethacrylate: 30%–<35% 1,6‐hexanediol dimethacrylate: 1%–<5% 2‐hydroxyethyl methacrylate: 1%–<5% Diphenyl(2,4,6‐trimethylbenzoyl)phosphine oxide: 1%–<5% Hydroxy propyl methacrylate: 1%–<5% Phenyl bis(2,4,6‐trimethylbenzoyl)‐phosphine oxide: <1%
Denturetec (AM‐S)	Additively manufactured denture base resin	Ethoxylatedbisphenol A dimethacrylate, Urethanmethacrylate, Triethylene glycol dimethacrylate, pyrogenic silica, catalysts, inhibitors, pigment

For additively manufactured specimens, a disk‐shaped standard tessellation language (STL) file was generated (Meshmixer v3.5.474; Autodesk Inc), transferred into nesting software (Composer; Asiga), and positioned with a 45‐degree angle to the build platform.^5^ After automatically generating supports, this configuration was duplicated 10 times and the specimens were printed with a layer thickness of either 50 µm (AM‐N‐50, AM‐F‐50, and AM‐S‐50) or 100 µm (AM‐N‐100, AM‐F‐100, and AM‐S‐100) by using a digital light processing printer (Max UV; Asiga). After fabrication, AM‐N specimens were cleaned in an ultrasonic bath containing ethanol for 3 min followed by thorough cleaning in an ultrasonic bath containing fresh ethanol for 2 min. Then, specimens were light‐polymerized by using the manufacturer's proprietary curing unit (NextDent LC‐3DPrint Box; NextDent B.V.) for 30 min at 60°C.^38^ AM‐F specimens were cleaned in an ultrasonic bath containing isopropanol for 3 min followed by thorough cleaning in an ultrasonic bath containing fresh isopropanol for 3 min.^39^ AM‐S specimens were cleaned by using isopropanol‐soaked cloths until the excess resin was removed.^40^ All AM‐F and AM‐S specimens were light‐polymerized by using a xenon‐light polymerization unit (Otoflash G171; NK‐Optik GmHb) for 4000 cycles (2 × 2000).^39^, ^40^ For subtractively manufactured specimens, a cylinder (Ø 10 mm) was designed in STL format by using the same software, and a 5‐axis milling unit (Milling unit M1; Zirkonzahn GmbH) was used to fabricate cylinders from prepolymerized CAD‐CAM disks. These cylinders were then wet‐sliced into disk‐shaped specimens of desired dimensions by using a precision cutter (Vari/cut VC‐50; Leco Corporation).

A non‐contact optical profilometer equipped with a CWL 300 µm sensor (FRT MicroProf 100; Fries Research & Technology GmbH) was used to record R_a_ values with the parameters of 5.5 mm of tracing length, 0.8 mm of cut‐off Lc value, 3 nm of z‐resolution, and a pixel density of 5501 point/line. Six linear traces (three horizontal and three vertical) that were 1 mm apart from each other were measured for each specimen and these values were averaged by using software (Mark III; Fries Research & Technology GmbH).^11^

A slurry of coarse pumice in water (Pumice fine; Benco Dental) was used to conventionally polish one surface of all specimens for 90 s at 1500 rpm after initial R_a_ measurements. Fine polishing was performed by using a polishing paste (Fabulustre; Grobet USA) for an additional 90 s.^41^ All polishing procedures were performed on a polishing box (Poliereinheit PE5; Degussa AG). All specimens were ultrasonically cleaned in distilled water for 10 min (Eltrosonic Ultracleaner 07–08; Eltrosonic GmbH), dried with paper towels, and R_a_ measurements were repeated.

A Vickers hardness tester (M‐400 Hardness Tester; Leco Corp) was used to measure the initial MH values. Each specimen was subjected to a load of 245 mN for 30 s^30^ at five different sites that were at least 0.5 mm apart from each other. These values were then averaged to calculate the final MH value of each specimen.

After MH measurements, color coordinates (L*, which corresponds to lightness; a*, which corresponds to redness; b*, which corresponds to yellowness) defined by the Commission Internationale de l'éclairage (CIE) were measured over a gray background by using a digital spectrophotometer (CM‐26d; Konica Minolta). This spectrophotometer has an illumination aperture of 8 mm and uses CIE D65 illuminant and the CIE Standard (2‐degree) human observer characteristics in its color estimations. Before each measurement, the spectrophotometer was calibrated in line with the manufacturer's recommendations, and a saturated sucrose solution was used for optical contact between the specimen and the background. All measurements were performed three times in a temperature‐ and humidity‐controlled room with daylight and these readings were averaged.

**FIGURE 1:**
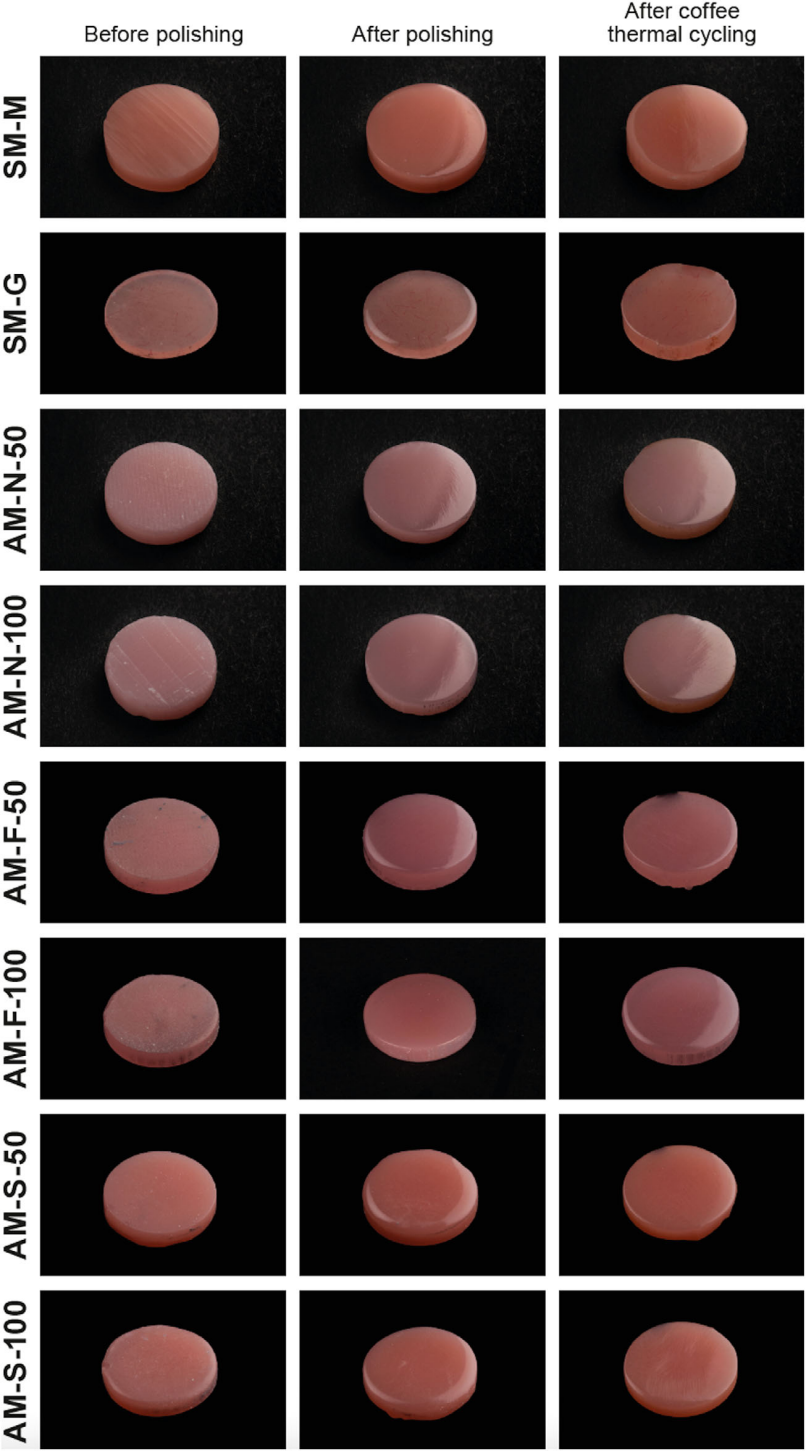
Representative images of specimens from each at different time intervals.

After initial measurements, all specimens were subjected to coffee thermal cycling for 5000 cycles (SD Mechatronik Thermocycler; SD Mechatronik GmbH) at 5°C–55°C with a dwell time of 30 s and a transfer time of 10 s.^8^, ^42^ A tablespoon of coffee (Intenso Roasted and Grounded; Kaffeehof GmbH) was dissolved in 177 mL of water to prepare the coffee solution, which was freshly made every 12 h by using a coffee machine.^8^, ^42^ After coffee thermal cycling, coffee extracts were cleaned by gently brushing the specimens 10 times with a toothpaste (Nevadent Mint Fresh; DENTAL‐Kosmetik GmbH) under running water (Figure 1).^8^, ^42^ R_a_, MH, and color coordinate measurements were repeated after coffee thermal cycling. A single operator (N.W.) performed all experiments and procedures in the present study. The CIEDE2000 formula with parametric factors (k_L_, k_C_, and k_H_), which are correction terms for variation in experimental conditions, set to one was used to calculate the color differences (ΔE_00_) among materials:^8^, ^42^

CIEDE2000=ΔL′/kLSL2+ΔC′/kCSC2+ΔH′/kHSH2+RTΔC′/kCSCΔH′/kHSHΔL′/kLSL2+ΔC′/kCSC21/2

Scanning electron microscopy (SEM) (LEO 440; Zeiss) images of one additional sample from all test groups were taken at each time interval (before polishing, after polishing, and after coffee thermal cycling) under ×50 magnification at 20 kV to analyze surface topography after coating the surface of the specimens with gold. Ra and MH data were evaluated by using a linear mixed effect model with material type, time interval, and the interaction between these factors as covariates. One‐way ANOVA was used to analyze the ΔE_00_ data. All analyses were performed by using software (SPSS v23; IBM Corp) at a significance level of α = 0.05. In addition, ΔE_00_ values were further evaluated by the previously set perceptibility and acceptability thresholds for denture base resins (perceptibility: 1.72, acceptability: 4.08).^27^

## RESULTS

The descriptive statistics of Ra values of each material‐time interval pair are summarized in Table 2. The generalized linear model showed that the interaction between the material type and the time interval was effective on both Ra and MH, along with significant effects of material type and time interval as main factors (*p* < 0.001). All materials had their highest Ra before polishing (*p* ≤ 0.029) and the differences between after‐polishing and after‐coffee thermal cycling values were nonsignificant (*p* ≥ 0.814). Before polishing, AM‐F‐100 had the highest Ra (*p* < 0.001) that was followed by AM‐N‐100 (*p* < 0.001). SM‐M and SM‐G had similar Ra (*p* = 0.069) that was lower than those of other groups (*p* < 0.001). AM‐N‐50, AM‐S‐100, and AM‐F‐50 had similar Ra (*p* ≥ 0.225) that was higher than that of AM‐S‐50 (*p* < 0.001). After polishing, SM‐G had lower Ra than all materials (*p* ≤ 0.036) other than SM‐M and AM‐S‐50 (*p* ≥ 0.121). Every other pairwise comparison was nonsignificant (*p* ≥ 0.157). After coffee thermal cycling, SM‐G had lower Ra than all materials (*p* ≤ 0.002) other than AM‐N‐50 and AM‐S‐50 (*p* ≥ 0.116). In addition, AM‐N‐100 had higher Ra than AM‐F‐50 (*p* < 0.001). The remaining pairwise comparisons were nonsignificant (*p* ≥ 0.120).

**TABLE 2:** Mean ± standard deviations (min‐max) of Ra (µm) values.

	Before polishing	After polishing	After coffee thermal cycling
SM‐M	0.39 ± 0.16^bA^ (0.16–0.70)	0.22 ± 0.21^aAB^ (0.08–0.8)	0.21 ± 0.04^aBC^ (0.15–0.26)
SM‐G	0.54 ± 0.10^bA^ (0.43–0.76)	0.10 ± 0.03^aA^ (0.06–0.15)	0.11 ± 0.03^aA^ (0.06–0.16)
AM‐N‐50	5.05 ± 1.13^bC^ (3.64–7.45)	0.30 ± 0.25^aB^ (0.15–1.04)	0.25 ± 0.25^aABC^ (0.12–0.97)
AM‐N‐100	9.66 ± 1.38^bD^ (7.51–11.80)	0.17 ± 0.04^aB^ (0.10–0.22)	0.23 ± 0.02^aC^ (0.19–0.27)
AM‐F‐50	5.94 ± 1.35^bC^ (3.72–8.33)	0.22 ± 0.09^aB^ (0.10–0.39)	0.19 ± 0.04^aB^ (0.14–0.25)
AM‐F‐100	15.60 ± 3.47^bE^ (10.10–20)	0.19 ± 0.11^aB^ (0.09–0.42)	0.20 ± 0.07^aBC^ (0.12–0.32)
AM‐S‐50	3.04 ± 0.47^bB^ (2.09–3.64)	0.17 ± 0.16^aAB^ (0.06–0.62)	0.15 ± 0.12^aABC^ (0.07–0.49)
AM‐S‐100	5.38 ± 1.49^bC^ (3.73–8.68)	0.19 ± 0.05^aB^ (0.14–0.31)	0.20 ± 0.04^aBC^ (0.13–0.27)

^*^Different superscript letters present significant differences (lowercase letters for rows, uppercase letters for columns) (*p* < 0.05).

After polishing, SM‐G had higher MH than the remaining materials (*p* ≤ 0.025) other than AM‐F‐50 (*p* = 0.115). AM‐F‐50 and SM‐M had higher MH than AM‐S, AM‐N, and AM‐F‐100 (*p* < 0.001). AM‐S‐100 had higher MH than AM‐F‐100, AM‐S‐50, and AM‐N‐50 (*p* < 0.001). After coffee thermal cycling, AM‐S‐50, AM‐F‐100, and AM‐N had similar MH (*p* ≥ 0.223) that was lower than that of other materials (*p* < 0.001). In addition, AM‐F‐50 and SM‐G had higher MH than SM‐M (*p* ≤ 0.004). Coffee thermal cycling reduced the MH of SM‐M (*p* < 0.001) and increased that of AM‐S‐100 (*p* = 0.024). However, it did not affect the MH of the remaining materials (*p* ≥ 0.063) (Table 3).

**TABLE 3:** Mean ± standard deviations (min‐max) of microhardness values.

	After polishing	After coffee thermal cycling
SM‐M	28.6 ± 1.5^bC^ (27–32.4)	25.7 ± 0.9^aB^ (24.3–27.6)
SM‐G	31.3 ± 2.8^aD^ (28–34.7)	29 ± 2.1^aC^ (25.6–32.3)
AM‐N‐50	22.1 ± 0.5^aA^ (21.1–22.9)	22.6 ± 0.7^aA^ (21.6–24)
AM‐N‐100	22.7 ± 0.5^aAB^ (22.1–23.7)	22.8 ± 0.4^aA^ (22.1–23.8)
AM‐F‐50	28.9 ± 2.7^aCD^ (26.3–35)	28.6 ± 2.2^aC^ (25.5–32.5)
AM‐F‐100	20.8 ± 2.5^aA^ (17.7–26.4)	22.2 ± 2.6^aA^ (19–28.1)
AM‐S‐50	21.2 ± 2.1^aA^ (17.8–24.5)	22.1 ± 1.6^aA^ (20.2–25.3)
AM‐S‐100	25.1 ± 2.3^aB^ (21.6–28.9)	27.4 ± 1.8^bBC^ (24.4–29.9)

^*^Different superscript letters present significant differences (lowercase letters for rows, uppercase letters for columns) (*p*<0.05).

One‐way ANOVA showed that the differences among ΔE_00_ values were significant (*p* = 0.019) (Table 4). AM‐N‐100 had higher ΔE_00_ than all materials (*p* ≤ 0.009) other than SM‐M (*p* = 0.074), AM‐S‐50 (*p* = 0.375), and AM‐N‐50 (*p* = 0.462). AM‐S‐50 and AM‐N‐50 had higher ΔE_00_ than AM‐F‐100, AM‐F‐50, and SM‐G (*p* ≤ 0.024). Every other pairwise comparison was nonsignificant (*p* ≥ 0.057).

**TABLE 4:** Mean ± standard deviations (min‐max) of ΔE00 values.

Materials	ΔE_00_ values
SM‐M	1.16 ± 1.05^abc^ (0.15–3.41)
SM‐G	0.63 ± 0.54^a^ (0.21–2.20)
AM‐N‐50	1.51 ± 0.74^bc^ (0.73–2.74)
AM‐N‐100	1.76 ± 1.12^c^ (0.49–3.40)
AM‐F‐50	0.69 ± 0.19^a^ (0.39–1.04)
AM‐F‐100	0.59 ± 0.25^a^ (0.23–1.18)
AM‐S‐50	1.46 ± 0.87^bc^ (0.78–3.61)
AM‐S‐100	0.87 ± 0.21^ab^ (0.63–1.23)

^*^Different superscript letters present significant differences (*p* <0.05).

**FIGURE 2:**
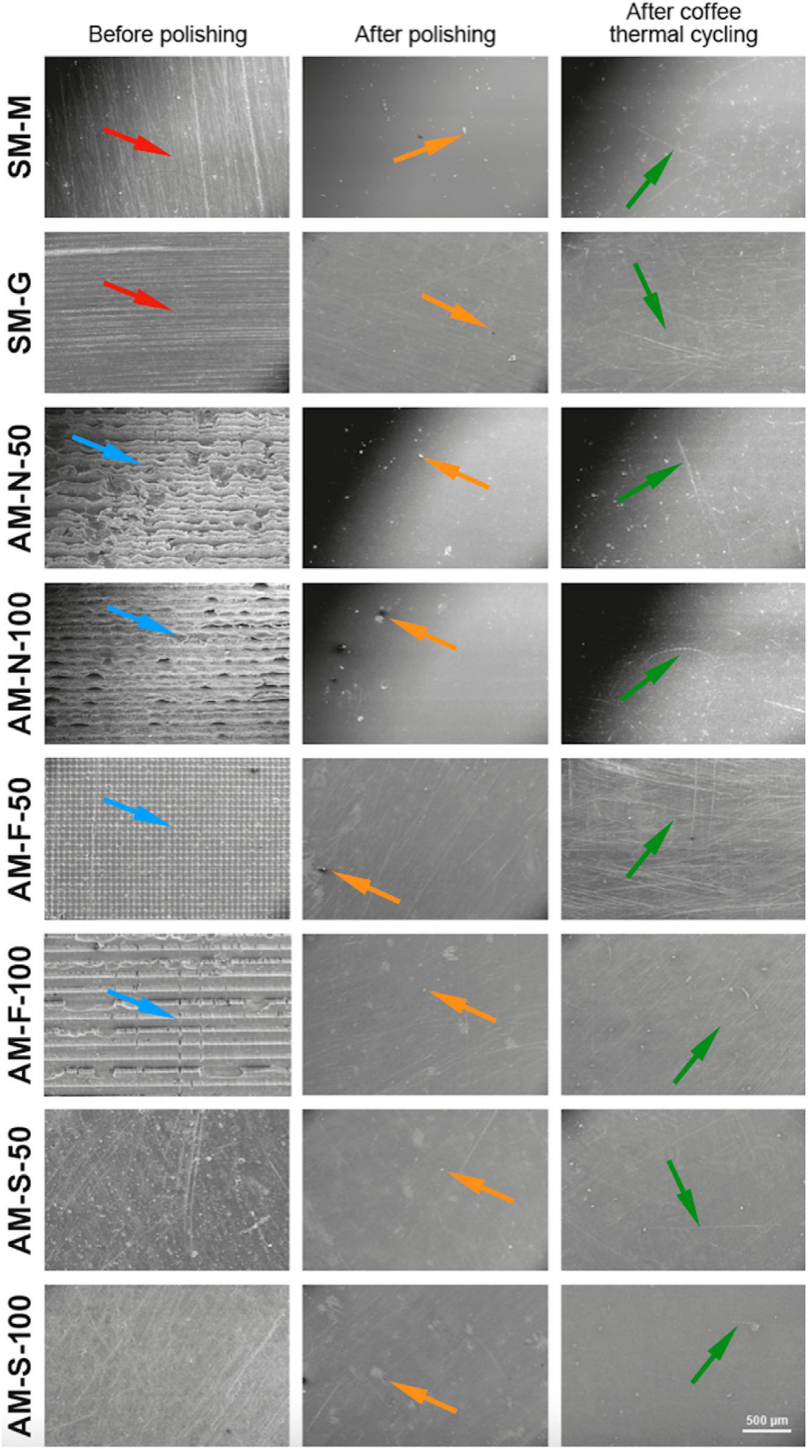
Representative SEM images (×50) of surfaces after different time intervals. Red arrow indicates longitudinal lines on subtractively manufactured specimens, blue arrows indicate lamellar structure of additively manufactured specimens, orange arrows indicate pores, and green arrows indicate complex small lines, SEM, scanning electron microscopy.

Regardless of the material tested, SEM images before polishing had prominent irregularities. However, the surface of SM‐M and SM‐G was characterized by longitudinal lines, whereas lamellae were dominant on AM‐N and AM‐F specimens. Polishing significantly smoothened the surface of all specimens and pores became visible, while complex small lines were visible on the surfaces after coffee thermal cycling (Figure 2).

## DISCUSSION

The first null hypothesis of the present study was rejected because the material type and time intervals affected the Ra of tested denture base materials. Regardless of the material, additively manufactured specimens with 100 µm layer thickness had higher Ra, which is in line with the results of a recent study that reported a similar trend when the specimens were fabricated at a 45‐degree angle.^13^ However, the differences in Ra within each material were nonsignificant after polishing and coffee thermal cycling. Before polishing, SM‐M and SM‐G had the lowest Ra, which could be associated with the fact that these specimens were fabricated by using prepolymerized PMMA disks that have lower residual monomer content and a higher degree of polymerization due to being fabricated under high pressure and high temperature.^21^ However, none of the test groups had Ra values that were either similar to or lower than the acceptable threshold of 0.2 µm before polishing. In line with previous studies, polishing significantly reduced these values,^5^, ^11^, ^17^, ^18^, ^23^ whereas coffee thermal cycling did not have a significant effect. When initial Ra values are excluded, the greatest difference with the clinically acceptable threshold belonged to AM‐N‐50 after polishing (0.30 µm). Considering that a quantitative difference of 0.1 µm is rather low, the authors believe this difference could be negligible. Nevertheless, this hypothesis needs to be corroborated by studies investigating the bacterial plaque accumulation on these surfaces.

Recent studies have also compared the Ra values of denture base materials tested in the present study.^17^, ^18^ In one of those studies, SM‐M, SM‐G, and AM‐F were evaluated, and Çakmak et al.^17^ showed that AM‐F had lower Ra than SM‐M after polishing. The layer thickness of AM‐F specimens was not disclosed by Çakmak et al.^17^ which complicates a direct comparison between studies. The other study investigated how different cleansing methods affected the Ra of SM‐M, SM‐G, AM‐N‐50, and AM‐S‐50, but also involved polishing's effect on Ra as a factor.^18^ The authors^18^ concluded that the Ra of test groups were similar after polishing, which contradicts the results of the present study. However, 30 specimens per group were tested in that study^18^ and the increased number of specimens may affect the results of this study, given the high standard deviation values of SM‐M, AM‐N‐50, and AM‐S‐50 when their mean Ra was considered (Table 2). While coffee thermal cycling did not affect the Ra values in the present study, previous studies contradict this finding.^8^, ^17^ Alp et al.^8^ evaluated the Ra of three subtractively, including SM‐M, and one conventionally manufactured denture base material, and showed that coffee thermocycling increased the Ra of all materials. In the other study^17^ mentioned above, the effect of coffee thermal cycling differed according to tested material and SM‐G was shown to have the lowest Ra after coffee thermal cycling. These contradicting results highlight the need for future studies involving longer durations of coffee thermal cycling on the Ra of additively and subtractively manufactured denture base materials as in all studies the specimens were subjected to 5000 cycles.

SEM images are parallel with the Ra results as all materials had the most irregular surfaces before polishing. The difference in the topography of the surfaces before polishing could be associated with the differences in manufacturing methods. Prominent longitudinal lines visible on the surface of SM‐M and SM‐G are possibly due to milling instruments and precision cutter, while the layer‐by‐layer manufacturing principle is clearly visible in the SEM images of additively manufactured specimens, particularly those of AM‐N and AM‐F. In addition, the higher number of layers of specimens with 50 µm layer thickness compared with those of 100 µm layer thickness is also apparent. Even though polishing led to smoother surfaces for all specimens, AM‐N‐50 had a higher number of pores than AM‐N‐100, which might be associated with the nonsignificantly higher Ra values after polishing. When after‐coffee thermal cycling SEM images were considered, AM‐N‐100 had more prominent surface deterioration, which might have been caused by the absorption of water, when compared with AM‐N‐50. Increased layer thickness might have diminished the bond between consecutive layers during the fabrication of specimens with 100 µm layer thickness and led to the nonsignificant increase in their Ra.

Regardless of time interval, SM‐G mostly had higher MH values than those of other materials and coffee thermal cycling reduced the MH of SM‐M and increased that of AM‐S‐100. Therefore, the second null hypothesis was rejected. When additively manufactured materials were further evaluated, AM‐F‐50 and AM‐S‐100 had higher MH than their counterparts in both time intervals, which could be interpreted as the effect of layer thickness on MH being material‐dependent as tested materials have different chemical compositions. These results contradict those of Lee et al., which showed that AM‐N‐50 had higher MH than AM‐N‐100.^14^ However, the specimens were polished by using a different methodology.^14^ and no thermal aging was performed. In addition, the MH of the other tested resin^14^ was not affected by the layer thickness, which corroborates the findings of the present study and the hypothesis on the effect of layer thickness on MH being material‐dependent. Even though the chemical composition of SM‐G is not disclosed by its manufacturer, it is the only nanographene‐reinforced material, which could be associated with its high MH values. The trend of high MH values was also observed with SM‐M, and standardized polymerization of subtractively manufactured denture base materials^21^ may again be related to this finding. Additively manufactured specimens mostly had lower MH values than those of subtractively manufactured specimens, which could be related to the residual monomers that negatively affect mechanical properties^34^ as these materials were post polymerized after fabrication. In addition, after coffee thermal cycling MH values of SM‐M were mostly higher than those of additively manufactured specimens after polishing. Therefore, it can be speculated that other than AM‐F‐50 and AM‐S‐100, tested additively manufactured denture base resins could be more prone to deformation intraorally even after SM‐M was subjected to long‐term consumption of coffee and intraoral thermal changes. It should also be noted that the decrease in the MH of SM‐M may be clinically negligible given that a universally accepted threshold value for hardness is not available. Also, longer exposures to thermal stresses might also diminish the MH of tested additively manufactured denture base resins.

Studies comparing the MH of additively manufactured denture base resins with other denture base materials are limited^4^, ^6^, ^10^, ^16^, ^30^, ^35^, ^43^ and only three of those studies involved subtractively manufactured denture base resins.^6^, ^16^, ^43^ In a recent study, Çakmak et al.^16^ concluded that AM‐S had lower MH than SM‐G after thermal cycling. In addition, thermal cycling decreased the MH of SM‐G in that study.^16^ However, a direct comparison between the present and Çakmak et al.^16^ may be misleading, given that coffee was not involved in that study and the layer thickness of AM‐S was not disclosed. Even though no aging was performed in their study, Fouda et al.^6^ have also reported parallel results to those of the present study. The authors^6^ concluded that subtractively manufactured denture base materials had higher MH than additively manufactured denture base materials, which also involved AM‐N‐50. However, the MH values of AM‐N‐50 in the present study were higher than those in Fouda et al.,^6^ which may be related to the differences in the printer used and the printing orientation. Prpić et al.^43^ stated that the additively manufactured denture base resin had higher Brinell's hardness than one of the tested subtractively manufactured denture base resins without any thermal cycling.

The third null hypothesis was rejected as significant differences in ΔE_00_ values were observed among tested materials. However, the differences between specimens fabricated with different layer thicknesses were nonsignificant within each additively manufactured material. In addition, among the test groups, only AM‐N‐100 had a mean perceptible color change as it had a slightly higher ΔE_00_ value (1.76) than the perceptibility threshold (1.72)^27^; thus, all materials had acceptable color stability. A recent study has investigated the stainability of a tooth‐colored additively manufactured resin when fabricated by using different layer thicknesses.^37^ The authors^37^ have concluded that specimens fabricated with 25 µm‐thick layers had significantly lower ΔE_00_ values than those fabricated with 100 µm‐thick layers, regardless of the immersion medium and duration. This contradiction between the present and Lee et al.^37^ may be associated with the differences in the resins tested. A recent study has also evaluated the stainability of SM‐M, SM‐G, and AM‐F after coffee thermal cycling.^17^ The authors^17^ concluded that only AM‐F had perceptible color change, while SM‐M and SM‐G had imperceptible color change.

A limitation of the present study was that only two‐layer thicknesses were compared. However, it is possible to change the layer thickness between 25 and 150 µm while using a photopolymerization 3D printer.^30^ In addition, additively manufactured specimens were fabricated with a standardized printing orientation. However, other studies have reported fabricating additively manufactured specimens with different angles and orientations may affect the results.^6^, ^11^, ^13^ Even though all additively manufactured specimens were fabricated by using a standardized printer that was also used in previous studies,^5^, ^17^ different printers with the same or different technologies may lead to different results. Subtractively manufactured specimens were wet‐sliced by using a precision cutter after milling cylinder‐shaped specimens from prepolymerized disks. This was deliberately preferred to limit the amount of excess material that would be generated to fabricate the specimens. However, this methodology does not replicate actual clinical situations and may be considered as a limitation. Coffee thermal cycling might have exacerbated the results of the present study as only polished surfaces of dentures are in contact with staining solutions clinically. Given that the main of the present study was to evaluate the effect of printing layer thickness on different properties of additively manufactured denture base materials, only one staining solution was used. Nevertheless, different staining solutions may lead to different results and the possible effect of saliva was not simulated in the thermal cycling setup. Finally, a conventional polishing procedure was performed in the present study to evaluate the polishability of tested materials as a secondary outcome. However, the efficiency of polishing may be affected by the operator or the polishing method. Even though the results of the present study indicated that tested subtractively manufactured denture base resin was more stable in terms of tested parameters and may be more resistant to mechanical and esthetic complications when compared with tested additively manufactured denture base resin, future studies should elaborate the results of the present study by testing other mechanical and optical properties of additively manufactured denture base resins with different layer thicknesses after being subjected to longer durations of aging or other possible stresses such as brushing and chemical disinfection to broaden the knowledge on their limitations.

## CONCLUSIONS

Polishing reduced the surface roughness of all materials significantly, whereas coffee thermal cycling did not significantly affect the surface roughness. Layer thickness of tested additively manufactured resins only affected the microhardness values as 50 µm layer thickness mostly led to higher microhardness. SM‐G mostly had higher microhardness, regardless of time interval. Coffee thermal cycling decreased the microhardness of SM‐M and increased that of AM‐S‐100. All materials had acceptable color stability. However, AM‐N‐100's color change after coffee thermal cycling was perceptible, considering published thresholds. Considering these results, tested nanographene‐reinforced PMMA may be the favorable material in the long‐term for the fabrication of removable dentures among those tested, while the preference of layer thickness should be made according to the additively manufactured denture base resin.

## CONFLICT OF INTEREST STATEMENT

The authors declare no conflict of interest. The authors do not have any financial interest in the companies whose materials are included in this article.
